# Compressed sensing in the far-field of the spatial light modulator in high noise conditions

**DOI:** 10.1038/s41598-021-97072-2

**Published:** 2021-08-31

**Authors:** Akhil Kallepalli, John Innes, Miles J. Padgett

**Affiliations:** 1 School of Physics and Astronomy, University of Glasgow, Glasgow, G12 8QQ UK; 2 Innovation and Technology Group, Leonardo MW Ltd., Edinburgh, EH3 9GL UK

**Keywords:** Optical physics, Techniques and instrumentation, Imaging techniques, Adaptive optics, Imaging and sensing

## Abstract

Single-pixel imaging techniques as an alternative to focal-plane detector arrays are being widely investigated. The interest in these single-pixel techniques is partly their compatibility with compressed sensing but also their applicability to spectral regions where focal planes arrays are simply not obtainable. Here, we show how a phased-array modulator source can be used to create Hadamard intensity patterns in the far-field, thereby enabling single-pixel imaging. Further, we successfully illustrate an implementation of compressed sensing for image reconstruction in conditions of high noise. In combination, this robust technique could be applied to any spectral region where spatial light phase modulators or phased-array sources are available.

## Introduction

Traditional digital imaging systems use a lens to form an image of the scene on a focal-plane detector array, with each element of the array recording the corresponding intensity of the image pixel. Single-pixel imaging systems are an alternative to this where the detector array is replaced by a spatial light modulator which applies a mask to the image and then a large-area, single-pixel detector subsequently measures the total optical power transmitted by this mask^1^. This measured power is proportional to the overlap integral between the imaged scene and the chosen mask. By measuring the overlap integral for a wide range of different masks, it is possible, using various algorithms, to infer the image of the scene. These single-pixel approaches are useful at wavelengths where detector arrays are expensive or problematic in other ways, or where the enhanced time resolution of the single-pixel detector gives other detection modalities^2^ such as depth-resolved imaging^3–5^, microscopy^6,7^, Raman imaging^8,9^, to name a few. Furthermore, by appropriate choices of mask and associated reconstruction algorithms, it is possible to implement compressed sensing techniques^10–12^ to recover a high-quality image in fewer measurements than a more traditional raster-scanned approach.

**Figure 1:**
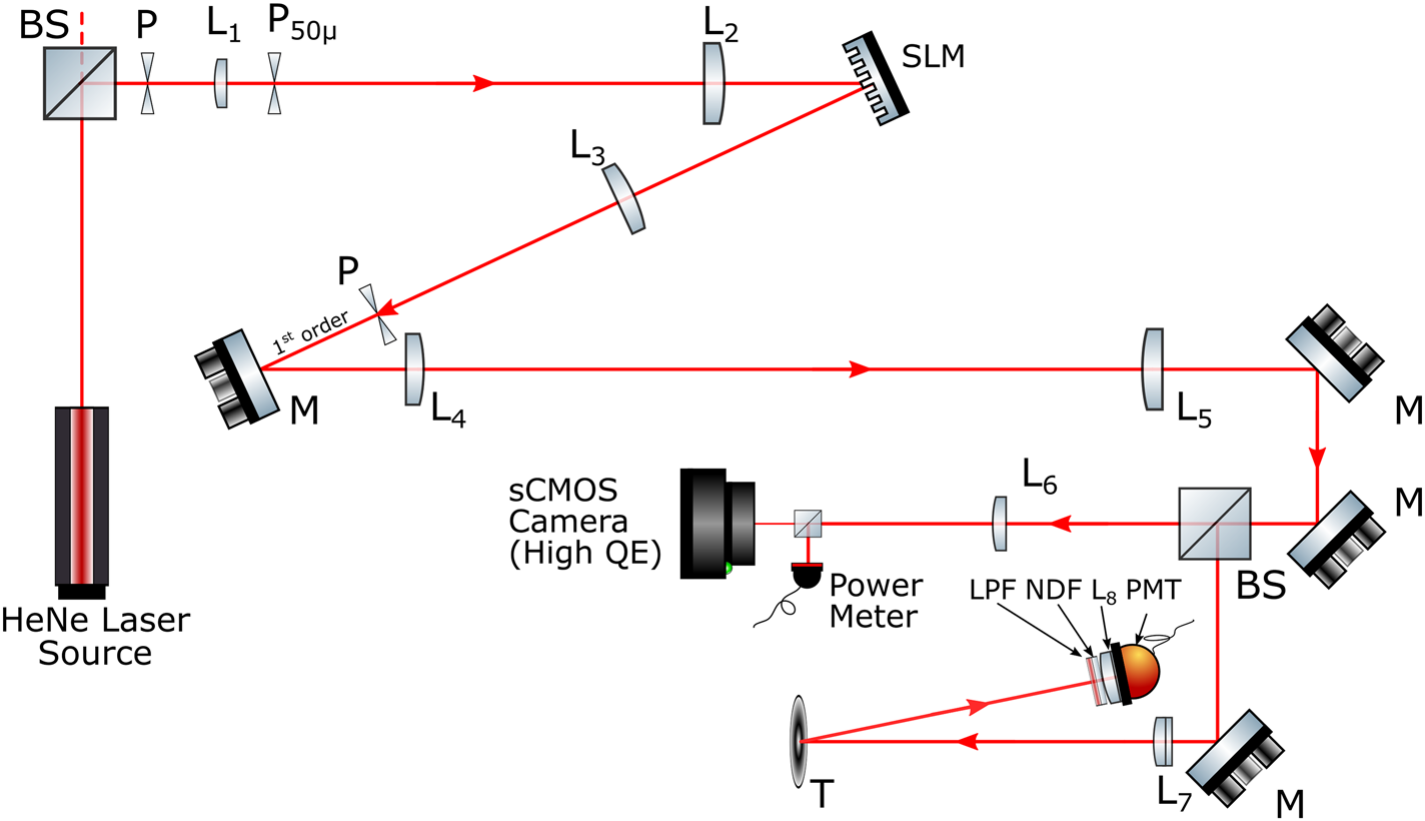
The output of the HeNe laser encounters a polarising beamsplitter that propagates the vertically polarised, reflected light in the direction of the spatial light modulator (SLM). The beam magnified and spatially filtered as it passes through a pinhole (P), a 50 mm focal length lens (L$_1$), a 50 $\mu$m precision pinhole (P$_{50 \mu }$) and a 400 mm focal length lens (L$_2$). The beam is modulated with Hadamard matrices displayed on a custom liquid-crystal SLM (Meadowlark Optics). The modulated light from the SLM is focused at an aperture (P) by 250 mm focal length lens (L$_3$) where the first order of diffracted light is selected. The light is subsequently propagated into the far-field through a 250 mm focal length lens (L$_4$) and a 400 mm focal length lens (L$_5$). The beam is propagated into two separate arms. One arm uses a power meter to measure the laser power and an ORCA-Flash CMOS Hamamatsu camera that images the illumination pattern. The second arm consists of the target (T) of which light is reflected towards an arrangement of a long pass filter (LPF), neutral density filters (NDF), an 80 mm focal length lens (L$_8$) and a Thorlabs photomultiplier tube (PMT, model PMM02), completing the single-pixel system.

**Figure 2:**
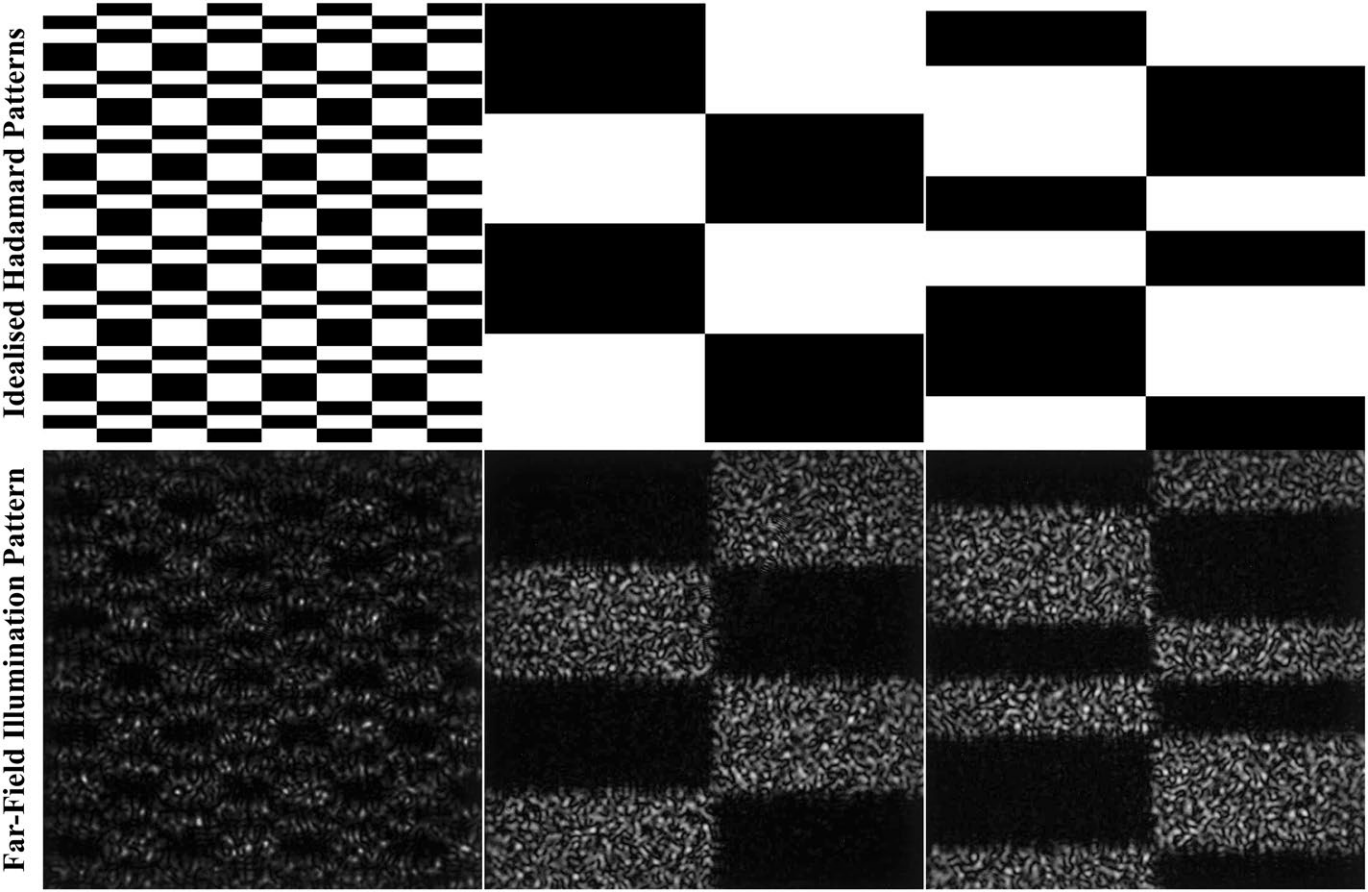
The Hadamard patterns (idealised) are projected in the far-field, and captured using a sCMOS camera (ref. Fig. 1) to illustrate the illumination distribution on the target

Single-pixel imaging systems can be configured in two distinct ways. In the first approach, the object can be flood (uniformly) illuminated and the backscattered light is imaged onto the plane of the spatial light modulator where the mask is applied. The reflected/transmitted light from the modulator is subsequently detected. Secondly, that same mask can be used to structure the illuminating light. The resulting backscatter of the target from the modulated light is measured by the detector directly. In this latter configuration, the approach has much in common with the technique commonly referred to as computational ghost imaging^13^. In both cases, choice of mask design and reconstruction algorithms are subject to the same considerations.

In many single-pixel imaging systems to date, the chosen basis for the masks has been the Hadamard basis due to it being a complete orthogonal set^14–16^. These masks are frequently implemented using a digital micro-mirror device (DMD) which works in reflection to apply binary intensity masks to the incident light with an update rate of less than 0.1 msec. However, inherent in the binary nature of the masks is that 50% of the light is lost. Another widely used spatial light modulator technology is based on a liquid crystal layer on a silicon backplane (LC-SLM) which can be electrically addressed to create spatially varying phase masks. Unlike the DMD, which is inherently binary in nature, the LC-SLM can typically create 128 different phase delays, in the 0-2$\pi$ range, for each effective pixel of the modulator. Although such a phase modulator can be combined with other optics to create a intensity modulator, the phase-only nature of the LC-SLM itself creates other opportunities for modulating the light.

Rather than creating a binary intensity mask in the image plane, the phase-only LC-SLM can act as a diffractive optical element. This allows the system to create computer generated holograms that can create chosen intensity distributions in the far-field. In principle, nearly all of the illumination light can be diffracted into the desired pattern, overcoming the 50% loss of the DMD. One approach for calculating the required spatial form of the required diffractive pattern encoded on the LC-SLM is the Gerchberg–Saxton (GS) algorithm^17^. The GS algorithm is applied in its original formulation to generate holograms for projection on the LC-SLM. We assess the suitability of the Hadamard pattern sources (idealised/hologram kinoform/imaged intensity) for those that contribute the least in terms of noise and/or uncertainty. This choice helps achieve the focus of this research optimally, i.e. to identify the best combination of individual elements for compressive sensing in poor SNR conditions.

In this work, we use the Gerchberg–Saxton algorithm to calculate the required spatial phase distributions that can be programmed on a LC-SLM to create Hadamard intensity patterns in the far-field. These Hadamard patterns are used to sequentially illuminate an object and the power backscattered by the object is measured. Knowing both the mask patterns and the measured power allows the image to be reconstructed. In addition, we also successfully illustrate compressed sensing with a selected subset of the pattern set to reconstruct the image, gaining the advantage of faster imaging and/or robustness in low signal-to-noise ratio (SNR) conditions.

We note here that although we demonstrate our approach in the optical regime, this approach is also suited to phased-array sources, such as those possible in the radar regime.

**Figure 3:**
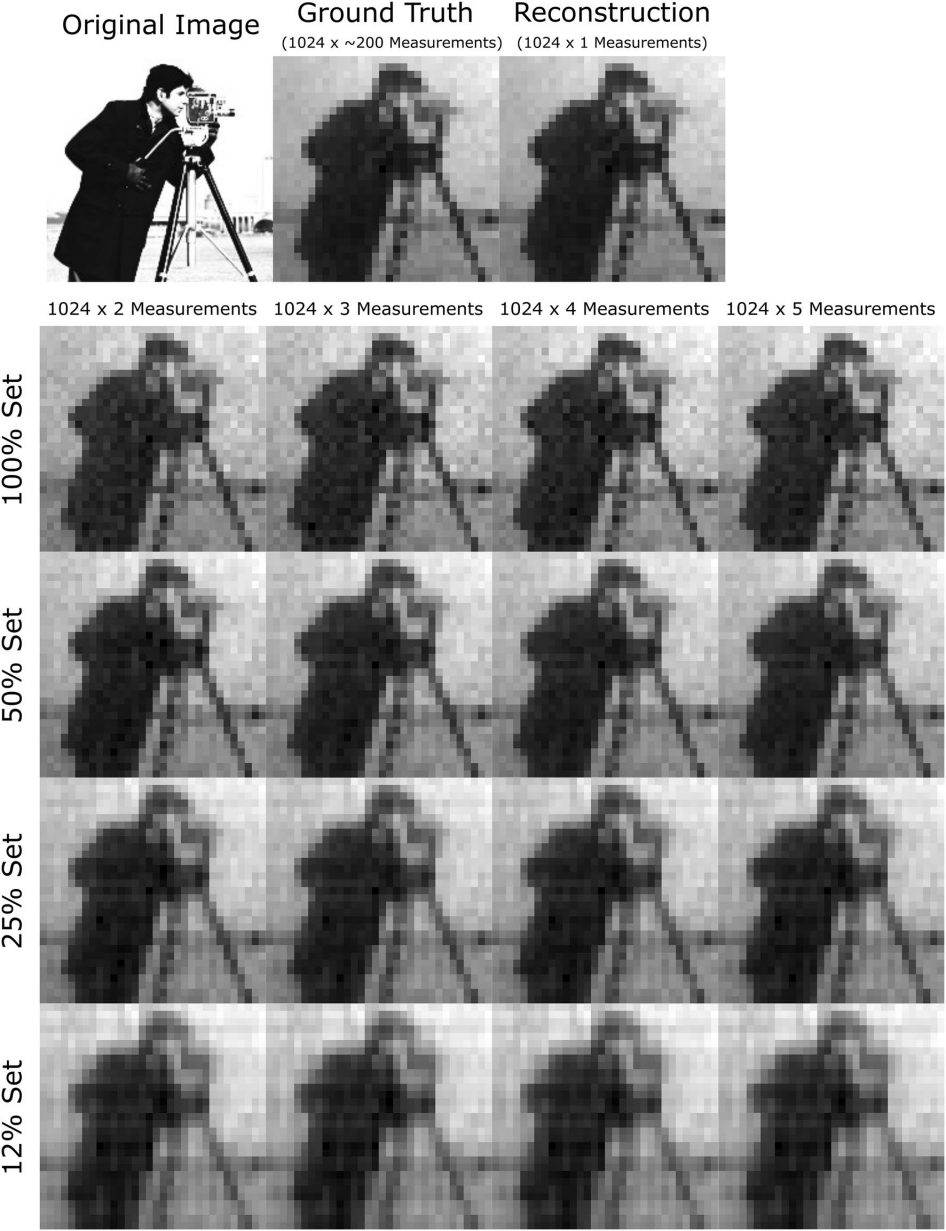
This illustration of compressed sensing uses the standard “cameraman” test image as an object in the experiment. The ground truth image obtained after $\approx$ 200 iterations of 1024 patterns is compared to reconstruction using 100%, 50%, 25% and 12% pattern subsets for up to 5120 measurements. The images here were acquired in a high SNR conditions corresponding to Fig. 4A. The “cameraman image” is adapted from the Massachusetts Institute of Technology, all rights reserved.

**Figure 4:**
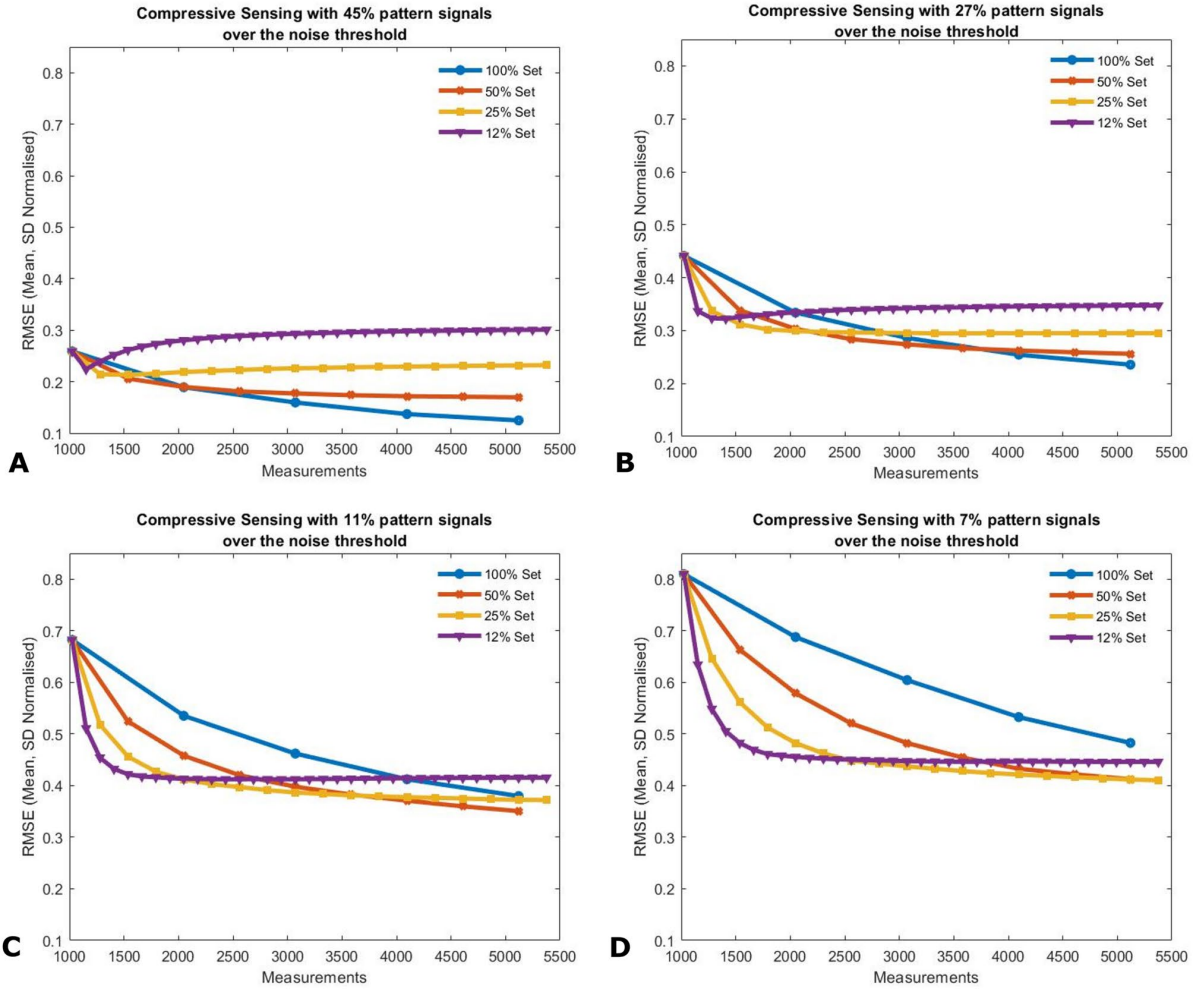
RMS errors of image reconstruction outputs are calculated with respect to the ground truth image as a function of the number of measurements for varying degrees of image compression and SNR regimes. The cases presented here are the ideal condition (**A**) and the three subsequent scenarios (**B**–**D**) with increasingly poor SNR. In low SNR conditions (**C**, **D**), compressed sensing shows a better reconstruction up until 3000–4000 patterns. However, the overall image quality does reduce as a function of SNR.

**Figure 5:**
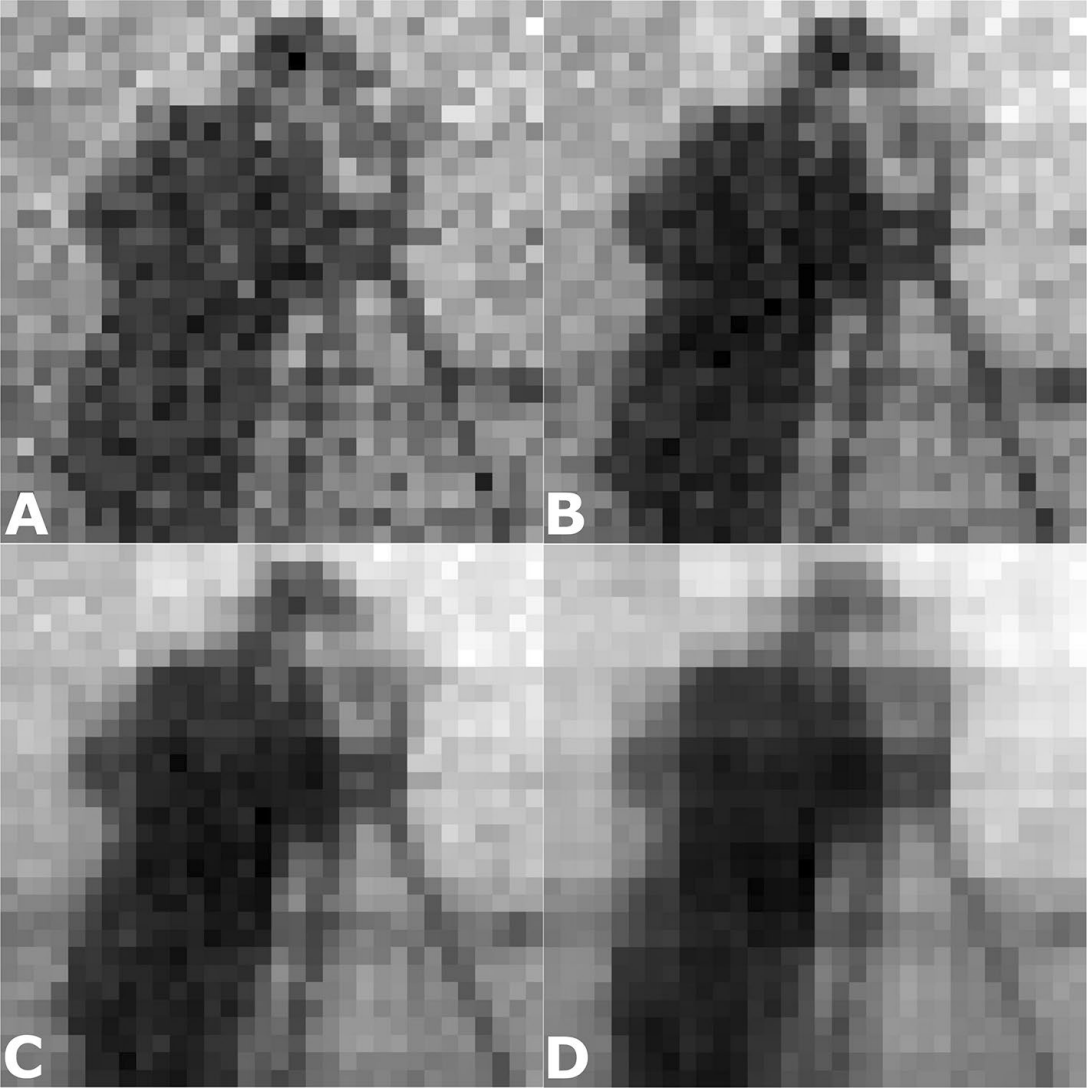
The images here are reconstructed using 2048 measurements in scenario shown in Fig. 4 (**C**). It illustrates a better reconstruction using a compressed sensing approach with 50% (**B**) and 25% (**C**) pattern sets in low SNR without any image processing. The “cameraman image” is adapted from the Massachusetts Institute of Technology, all rights reserved.

## Methods and results

As illustrated in Fig. 1, the output beam of the He-Ne laser fills the aperture of the spatial light modulator (SLM). The SLM is programmed to modulate the beam with the necessary diffractive optic kinoform, as calculated using Gerchberg–Saxton algorithm^17^, to create the Hadamard patterns in the far-field plane.

Originally, the GS algorithm was developed as an iterative solution for spatial phase structure in cases where the intensity in the diffraction and imaging planes are both defined. In our case, the known intensities are the profile of the laser beam used to illuminate the SLM and the desired intensity distribution in the far-field. Using the relations devised by Gerchberg and Saxton^17^, the phase mask for the SLM is calculated and superposed onto the incident beam. Once encoded on the SLM, the hologram is propagated by fast Fourier transformation and plane wave decomposition to simulate the propagation through the optical system (as detailed in Fig. 1) to be used for image reconstruction. Figure 2 shows instances of the ideal Hadamard pattern and the corresponding, real far-field intensity distribution as imaged by the sCMOS camera for comparison. Clearly, laser illumination results in speckle noise in the far-field as seen in the images captured by the sCMOS camera. When the images from the camera that have speckle noise are used in the reconstruction, the final reconstruction is visibly poorer. The choice of the pattern source is motivated by the least contribution of noise, which comes from the idealised patterns. Furthermore, each Gerchberg–Saxton derivation of our kinoform is seeded with a different random start giving different speckle pattern for each iteration of patterns such that the speckle noise is consequently suppressed.

As the modulator is used in a diffractive mode, a phase-grating is added to the kinoform so that the resulting Hadamard pattern is shifted to the first-order where it is selected from the other diffracted orders in the Fourier-plane with an aperture. The chosen order is propagated through a series of lenses into the far-field and into two optical paths—one with an sCMOS camera and the other with a single-pixel imaging system (photomultiplier tube, PMT). The PMT is positioned to collect the light backscattered from the object. This measurement is proportional to the overlap integral between the projected Hadamard pattern and the object. The sCMOS camera plays no role in the image acquisition but is included as a diagnostic tool to confirm the far-field illumination pattern. The overall experimental system is similar to that reported in Toninelli et al.^18^).

Although in principle, we could use GS to generate any orthogonal pattern basis, here we choose to use the Hadamard basis (instead of others such as the Fourier basis) since the application of this basis to compressed sensing has been heavily studied and understood. Additionally, the advantages of Hadamard patterns are assessed and detailed in a comparison with the Fourier-basis by Zhang et al.^16^. The Hadamard matrices used in this research are 32-by-32 square arrays of binary patterns, forming a complete orthonormal set of 1024 patterns. Historically, Hadamard matrix-based image encoding was developed to decompose an image into its Hadamard transforms as a method of image compression for reducing the bandwidth required for data transmission^14,15^. As natural images have a sparse representation in the Hadamard basis, measurement in this basis allows sharp image reconstruction with fewer measurements than pixels, a form of compressed sensing. In the experiment, the maximum spatial frequency of the Hadamard intensity patterns is limited in accordance with the diffraction limit. The scale of the Hadamard pattern is set accordingly to not exceed this limit.

Using a sequence of $i = 1 \ldots 1024$ 32-by-32 patterns to illuminate the target, we measured the corresponding signal from the photomultiplier tube (PMT). If the Hadamard patterns are described as $p_i (x,y)$ and the corresponding signals detected are $s_i$, then using traditional reconstruction algorithms (Eq. 1), we can reconstruct the image *I* from 1024 patterns^19^:

1 $$\begin{aligned} I = \sum _{i=1}^{N} (s_i - \langle s_i \rangle )p_i(x,y) \end{aligned}$$

where $\langle s_i \rangle$ is the average value of all single-pixel detector signals.

Initially, we considered two approaches to image reconstruction. To create an image, we used the signals measured by the PMT and the knowledge of (1) the ideal Hadamard patterns and (2) calculated holograms (which were generated using the iterative GS calculations and then propagated by plane-wave decomposition to the object plane). Initial testing showed a better image reconstruction and contrast using the measured signals and the idealised patterns, and therefore, became the choice for all subsequent image reconstruction. This also led to the advantage of not requiring additional computational resources, as would be needed for modelling the propagation from the SLM to the far-field.

In ideal conditions, with unlimited resources and time, reconstruction of the object can be done over innumerable iterations of the full pattern set, averaging the individual images to overcome noise. Indeed, we established a “ground truth” image using precisely this approach, averaging $\approx$ 200 iterations of the full pattern set, after which no further significant changes to the average image were apparent. This ground truth is then used as the comparison metric for all measurement scenarios in the experiment.

In practical situations, a more realistic scenario is that of a higher noise level and with constraints on the number of measurements that can be obtained within a given time or total illumination dose. For such cases, compressed sensing (utilising a subset of patterns that offer the greatest value to the reconstruction) is an option. Different approaches to compressed sensing have been illustrated in the past such as complementary compressive sensing in a telescopic system^20^ and single-pixel imaging in the near-infrared wavelengths for microscopy^21,22^. The compressive sensing method in the telescopic system uses both arms to reduce the sampling rate and achieve high resolution image reconstruction. The disadvantages, however, could be seen when atmospheric turbulence induces noise in the measured signal. Similarly, Denk et al.^22^ showed compressed sensing applied for single-pixel based microscopy using multiple, balanced Germanium photodiodes. In comparison, our method detailed here uses a single PMT for signal measurement and remains robust in poor SNR conditions (although tested in a laboratory setting). The compressed sensing is implemented using only a subset of the full pattern set to reconstruct the image. The subset itself is chosen based on the contribution of the pattern to the image reconstruction. This is quantified in terms of the measured signal strength. Note that this approach places no reliance upon image libraries or image priors and can be applied to arbitrary images.

Given a fixed number of measurements, the resulting image reconstruction can be enhanced by concentrating these measurement on the subset of the patterns most significant to the image. In our investigations we initiate each image acquisition using a full measurement set of patterns and then use the signals from this set to establish which subset of patterns are most important for defining the image. We then use the remaining measurement resource to repeatedly measure the signals and hence suppress the noise for these important patterns. For example, the same number of measurements required for a further 5 iterations of the full pattern set can instead be used for 20 iterations of the 25% set. After every iteration, to measure the quality of the images reconstruction we calculated the RMS error (Eq. 2) of the reconstruction compared to the ground truth image.

2 $$\begin{aligned} \begin{aligned} I_{norm}&= \frac{I - \mu }{\sigma } \\ RMSE&= \sqrt{\frac{1}{m} (I_{norm} - I_{GT,norm})^2} \end{aligned} \end{aligned}$$

where mean ($\mu$) and standard deviation ($\sigma$) of pixel values are used to normalise the reconstructed image and the RMS Error is calculated with the normalised ground truth ($I_{GT,norm}$) of the images of *m* pixels.

The compressed sensing scenarios were evaluated with various fractions of the the full pattern set and SNR constraints. Figure 3 shows the image reconstruction when using pattern sets with differing degrees of selectivity, for up to 5120 measurements in different experimental runs.

In parallel to the pattern set selectivity, we introduced SNR constraints with 4 scenarios, and characterised the resulting image quality in terms of a root-mean-square error, as shown in Fig. 4. Of the four cases, the first scenario illustrates a near ideal case with a high SNR. In this case, we observed that compressed sensing offers no advantages in the quality of the image irrespective of the number of measurements. The best images obtained are in the case of using the full pattern set (Fig. 4A). Thereafter, three scenarios of increasingly lower SNR were evaluated. We clearly observe that given a fixed number of measurements, lower RMS errors can be obtained by adopting a compressed sensing approach, repeatedly measuring only the most significant subset of patterns. For example, we see that a significant improvement in the image quality and lower RMS error when comparing the images after 2048 measurements in scenario C (Fig. 4) compared to measuring the full pattern set. The images corresponding to this specific SNR scenario after 2048 measurements for various degrees of pattern selectivity are shown in Fig. 5.

Obviously, the precise details of an optimum compressed sensing strategy depend critically upon on the SNR, alongside the nature of the object and what additional image priors might be assumed. In this work, we use no image priors and produce results without any image processing involved. Our result illustrates two key outcomes; (1) that the quality of our patterns formed in the far-field of the modulator allow us to recreate the scene from backscattered light and (2) the method’s robustness to facilitate a compressed sensing approach that yields an image improvement in high noise conditions.

## Conclusions

We have shown a successful implementation of single-pixel imaging using a phase-only modulator to create Hadamard intensity patterns in the far-field of the modulator. Using these patterns to illuminate the object, we measure the backscattered light and show image reconstruction through an iterative process under ideal conditions of complete measurement sets and high SNR. We also confirm the advantages of compressed sensing in achieving lower image RMS error and its applicability in lower SNR conditions. This approach to single-pixel imaging using spatial phase modulators to create Hadamard intensity patterns in the far-field is applicable to any imaging system where a spatial phase modulator or phased array source is available, including potentially in the radio frequency regime.

The data underlying the results presented in this paper (i.e. PMT signals, idealised Hadamard patterns) are accessible in the following repository^23^.
